# *Neisseria gonorrhoeae* Infection Induces Altered Amphiregulin Processing and Release

**DOI:** 10.1371/journal.pone.0016369

**Published:** 2011-01-27

**Authors:** Sonja Löfmark, Nele de Klerk, Helena Aro

**Affiliations:** Department of Genetics, Microbiology, and Toxicology, Stockholm University, Stockholm, Sweden; Columbia University, United States of America

## Abstract

Adhesion of the human pathogen *Neisseria gonorrhoeae* has established effects on the host cell and evokes a variety of cellular events including growth factor activation. In the present study we report that infection with *N. gonorrhoeae* causes altered amphiregulin processing and release in human epithelial cells. Amphiregulin is a well-studied growth factor with functions in various cell processes and is upregulated in different forms cancer and proliferative diseases. The protein is prototypically cleaved on the cell surface in response to external stimuli. We demonstrate that upon infection, a massive upregulation of amphiregulin mRNA is seen. The protein changes its subcellular distribution and is also alternatively cleaved at the plasma membrane, which results in augmented release of an infection-specific 36 kDa amphiregulin product from the surface of human cervical epithelial cells. Further, using antibodies directed against different domains of the protein we could determine the impact of infection on pro-peptide processing. In summary, we present data showing that the infection of *N. gonorrhoeae* causes an alternative amphiregulin processing, subcellular distribution and release in human epithelial cervical cells that likely contribute to the predisposition cellular abnormalities and anti-apoptotic features of *N. gonorrhoeae* infections.

## Introduction

The human pathogen *Neisseria gonorrhoeae* (gonococcus), the causative agent of the sexually transmitted disease gonorrhea, primarily colonizes the mucosal surface of the male urethra and the female cervix but also colonizes the vagina, pharynx, rectum and conjunctiva of the eye. Initial attachment of the bacteria to the apical side of epithelial tissues is mediated by type IV pili [1], [2], [3], [4]. The adherence mediates host cell signaling events and elicits a multitude of cellular responses, including cortical plaque formation [3], release of intracellular calcium [5], [6] and anti apoptotic factors [7], [8].

Also, 24 hours of *N. gonorrhoeae* infection alters cell cycle progression by the reduction of cyclin B1 levels in HeLa cells, causing early G1 arrest [9]. In G1 phase, growth factors, such as amphiregulin, are active to stimulate cellular growth and cell cycle progression.

Amphiregulin is a membrane-anchored glycoprotein, belonging to the epidermal growth factor (EGF) family and promotes a bi-functional role by stimulating growth of most cell types including normal epithelial cells as well as malignant cells while at the same time it inhibits the growth of certain aggressive carcinoma cell lines [10], [11], [12], [13]. Amphiregulin is synthesized as a pro-peptide and cleaved at the plasma membrane by metalloprotase ADAM17 giving rise to several different forms of the protein with varying sizes, cellular localizations and functions [11], [14], [15], which seems to depend on cell line as well as external induction of cells and growth conditions [15], [16]. The pro-amphiregulin contains several domains, including the bioactive part with a heparin binding domain and an EGF like domain, responsible for binding to the receptor [10]. The action of amphiregulin is mediated mainly by binding to the epidermal growth factor receptor (EGFR) also known as ErbB1 or HER1. The binding to the receptor on its own cell plasma membrane initiates a positive feedback loop of growth stimulation as well as it initiates a cascade of events leading to the expression of genes involved in cell cycle progression, cell growth and apoptosis resistance [17], [18], [19]. Amphiregulin also has the ability to directly interact with DNA and heterochromatin, and thereby potentially alter global gene transcription [10], [15].

Amphiregulin is also involved in bacterial infections. Host cell transcriptional upregulation of amphiregulin occurs in infections of *Shigella flexneri*, EHEC, and *Helicobacter pylori* [20], [21], [22]. Plant *et al.* showed that *Neisseria gonorrhoeae* causes a 20-fold upregulation of amphiregulin upon 2 hours of infection [23]. In addition, peptides derived from amphiregulin have been shown to possess antimicrobial activity against several pathogens in vitro [24].

In the present study we investigated amphiregulin in prolonged infection by *N. gonorrhoeae* in the human cervical epithelial cell line Me-180. We show that *N. gonorrhoeae* up-regulates gene transcription of amphiregulin. Further, the proteolytic cleavage pattern of amphiregulin at the plasma membrane is changed and the majority of induced amphiregulin is released in the cell supernatant, followed by a co-localization with the adhered bacteria at the plasma membrane.

## Results

### *N. gonorrhoeae* increases amphiregulin mRNA

**Figure 1:**
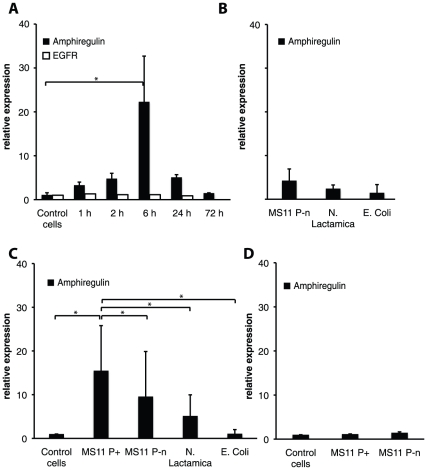
Amphiregulin is upregulated during *N. gonorrhoeae* infection. Relative amphiregulin mRNA expression was quantified by qPCR. Relative increase of mRNA in infected cells is presented, after normalization to the reference gene GAPDH. The black bars represent an average of three independent experiments and standard deviations of relative amphiregulin mRNA expression (* *P*<0.05). (**A**) Quantification of amphiregulin and EGFR mRNA in Me-180 cells at 1, 2, 6, 24, and for amphiregulin and additional time point at 72 hours post infection. The graph shows the relative change in mRNA levels of Me-180 cells infected with *N. gonorrhoeae* at different time points. The relative changes in EGFR mRNA are illustrated by the white bars and represent one single infection for each time point (**B**) Relative change in mRNA levels of Me-180 cells infected with *N. gonorrhoeae* MS11P-n, *N. lactamica* and *E. coli* at 6 hours of incubation (**C**) Relative change in mRNA levels of HEC-1-B cells infected with *N. gonorrhoeae* MS11 P^+^ and MS11P^−^n, *N. lactamica* and *E. coli* for 6 hours. (**D**) Relative change in mRNA levels of VK2/E6E7 cells infected with *N. gonorrhoeae* MS11 P^+^ and MS11P^−^n after 6 hours of incubation. The black bars represent an average of two independent experiments and standard deviations of relative amphiregulin mRNA expression.

Non-confluent epithelial like cervical Me-180 cells were infected with the piliated strain *N. gonorrhoeae* MS11 P^+^ for 1–72 hours. Total cell RNA was extracted, and cDNA was synthesized using eukaryotic-specific oligo-dT primers. Using real time quantitative polymerase chain reaction (qPCR) with amphiregulin gene specific primers, we measured the mRNA levels of amphiregulin and the amphiregulin receptor EGFR in Me-180 cells. An upregulation of the transcription of amphiregulin mRNA was detected after infection compared to untreated cells and normalized to housekeeping gene GAPDH. Increased mRNA levels were seen already after 1 hour with amphiregulin mRNA more than 3-fold upregulated. The upregulation peaked after 6 hours of infection with more than 20-fold increase in amphiregulin mRNA levels (Figure 1A). No change in transcriptional level was seen for the EGFR receptor 1–24 hours of infection, as compared to uninfected control cells (Figure 1A). A minor upregulation was also seen after 6 hours of infection with the non-piliated *N. gonorrhoeae* strain MS11 P^−^n and *N. lactamica* but not with *E. coli* (Figure 1B). This data suggests that the upregulation is specific for pathogenic *Neisseria* and that major increased transcription of amphiregulin is associated with bacterial adherence to host cells. The upregulation was also seen in HEC-1-B cells, with a 15-fold upregulation of amphiregulin mRNA after 6 hours of gonococcal infection but not after infection with *N. lactamica* or *E. coli* (Figure 1C). No change in amphiregulin mRNA levels was detected in vaginal epithelial VK2/E6E7 cells after gonococcal infection (Figure 1D).

### Bacterial infection induces alternative cleavage of amphiregulin and the product is released into the cell supernatant

**Figure 2:**
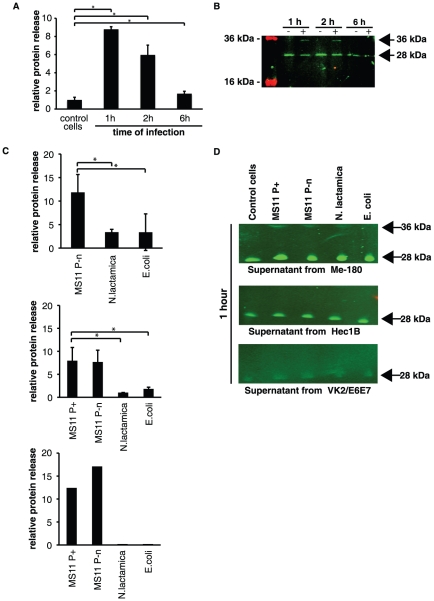
*N. gonorrhoeae* infection induces the release of a 36 kDa product of amphiregulin. Cell culture supernatants from infected cells and control cells were used to detect released amphiregulin in ELISA and Western blot. Supernatant was separated by SDS-PAGE and immunoblotted. By using polyclonal antibodies against amphiregulin (MBS220633), followed by secondary IgG antibodies conjugated with IRDye 800cw, the release of the 28 kDa domain was visualized. An additional band of 36 kDa in infected samples was identified and correlates to the alternative cleavage pattern exclusively induced by the infection. (**A**) Graph shows an ELISA assay of the release of amphiregulin into the Me-180 cell supernatants after infection with *N. gonorrhoeae* MS11 P^+^. The graph represents an average of two independent experiments and standard deviations (* *P*<0.05). (**B**) Western blots of Me-180 cell supernatants from 1, 2, and 6 hours incubations in the presence (+) or absence (−) of *N. gonorrhoeae* MS11 P^+^. (**C**) Graphs show the relative release of amphiregulin into the cell supernatants of Me-180 cells infected for 1 hour with MS11 P^−^n, *N. lactamica* and *E. coli* (upper left graph), HEC-1-B supernatants of cells infected for 1 hour with indicated strains (upper right graph). The graphs represent an average of two independent experiments and standard deviations (* *P*<0.05). Lower graph shows results from one ELISA of supernatants from VK2/E6E7 cells infected for 1 hour with indicated bacterial strains. (**D**) Western blots of ME-180 cell supernatants (upper membrane), HEC-1-B cell supernatants (middle membrane), and VK2/E6E7 cell supernatants (lower membrane) from 1 hour of incubation in the presence of different bacterial strains.

Since mRNA of amphiregulin was upregulated several fold during infection with *N. gonorrhoeae*, we then analyzed the protein expression, cleavage and release upon infection. Under normal conditions the full-length pro-amphiregulin is cleaved by metalloproteases at the plasma membrane and the bioactive domain of amphiregulin is released in the cell supernatant. To investigate whether an enhanced release could be seen upon infection with *N. gonorrhoeae,* cell culture supernatants from *N. gonorrhoeae* MS11 P^+^ infected Me-180 cells were collected 1, 2 and 6 hours post infection. The supernatant was used for coating wells over night for ELISA assay. A polyclonal antibody against the bioactive part of amphiregulin was used (MBS220633). Indeed, as shown in figure 2A there is an increase in amphiregulin protein in the medium already after one hour of infection with a more than eight fold increase compared to uninfected samples.

To further study the shedded proteins, the supernatant samples were analyzed by western blotting with the same antibody. The increased protein levels seen in figure 2A consisted of two different cleavage products of amphiregulin. The 28 kDa band represents the regular release of amphiregulin and did not increase upon infection. However, at one and two hours of infection an additional band at 36 kDa appeared (Figure 2B). This additional amphiregulin cleavage product is bigger in mass, indicating that amphiregulin processing induced by the infection is different from regular amphiregulin cleavage in uninfected control.

Also, non-piliated *N. gonorrhoeae* MS11 P^−^n caused an increased release of amphiregulin, suggesting that this high release is not dependent on bacterial adherence, but is *N. gonorrhoeae-*specific. Further, this is also attributed for HEC-1-B cells and VK2/E6E7 cells. Infection with non-pathogenic gram-negative bacteria resulted in a lower relative protein release after 1 hour (Figure 2C).

As seen in the western blot, the 36 kDa band released after one hour from Me-180 cells was specific for *N. gonorrhoeae* MS11 P^+^ and *N. gonorrhoeae* MS11 P^−^n infections, while no bands were visible in *E. coli* infected ME-180 cells (Figure 2D). The 36 kDa product was not visible after 1 hour of infection in HEC-1-B cells or VK2/E6E7 cells. However, HEC-1-B cells released very low levels of the alternatively processed form of amphiregulin after 6 hours of infection with MS11 P^+^ (Figure S1). In supernatant of uninfected cells, the minor release of amphiregulin was close to the level of the background signal. The controls with only bacterial suspension gave no signal, confirming that the products released are not of bacterial origin.

Taken together, these experiments show that during *N. gonorrhoeae* infection amphiregulin is cleaved off from the plasma membrane and a 36 kDa product is released into the extracellular media.

### The 36 kDa product of amphiregulin is binding to the plasma membrane and co-localizes with the bacterial adherence

**Figure 3:**
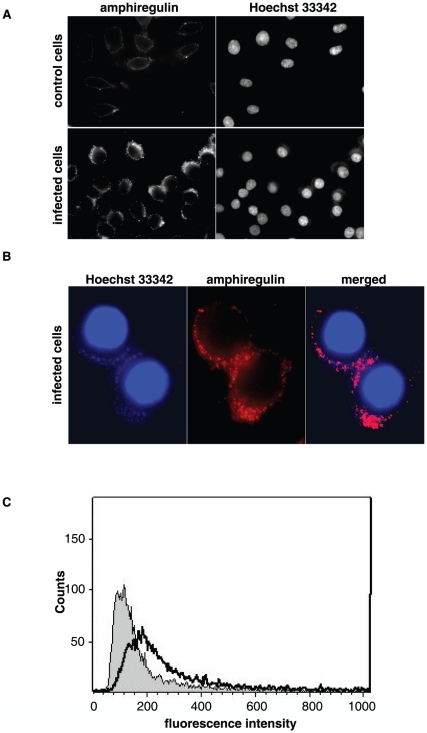
*N. gonorrhoeae* infection causes an increase of membrane bound amphiregulin that co-localizes with the site of bacterial adherence. Non-permeabilized Me-180 cells were infected with *N. gonorrhoeae* for 6 hours. Amphiregulin was detected by using polyclonal antibodies against amphiregulin (MBS220633), followed by secondary IgG antibodies conjugated with fluorofor Alexa 488 nm. (A) Representative immunofluorescence images visualizing the protein levels of amphiregulin on the plasma membrane in control cells and infected cells. Cellular DNA and bacterial DNA was stained with the cell-permeable dye Hoechst 33342. (B) High magnification images of infected Me-180 cells. Image showing the co-localization between surface bound amphiregulin (red) and bacterial DNA staining (blue). (C) Flow cytometry histogram showing the protein levels of plasma membrane bound amphiregulin in non-permeabilized cells. Amphiregulin staining on uninfected control cells is shown in grey while staining of infected cells is shown with a black line. The histogram is representative of two independent experiments.

We investigated the fate of the released products from the supernatant during infection. Me-180 cells were infected for 6 hours with *N. gonorrhoeae* MS11 P^+^ and fixed but not permeabilized, resulting in solid plasma membrane bound antibodies against the bioactive domain of amphiregulin (using antibody MBS220633). As compared to the uninfected control cells, the infected cells possess increased plasma membrane staining in immunofluorescence assay (Figure 3A). In addition, the fluorescent staining of amphiregulin co-localizes to the site of bacterial adherence, as determined by Hoechst 33342 staining of bacterial DNA (Figure 3B).

To quantify the increased plasma membrane binding of amphiregulin, 10 000 cells were counted in flow cytometry assay using the polyclonal antibody against the bioactive part of amphiregulin (MBS220633). Data from the flow cytometry analysis of surface bound protein further show an increase of cells positive for amphiregulin (Figure 3C). Since the release of amphiregulin is composed of two products, a 28 kDa bioactive domain, and a bacterial induced 36 kDa product, it is reasonable to assume that the increased amphiregulin staining seen is due to the 36 kDa product.

### *N. gonorrhoeae* changes the subcellular distribution of the bioactive part of amphiregulin

**Figure 4:**
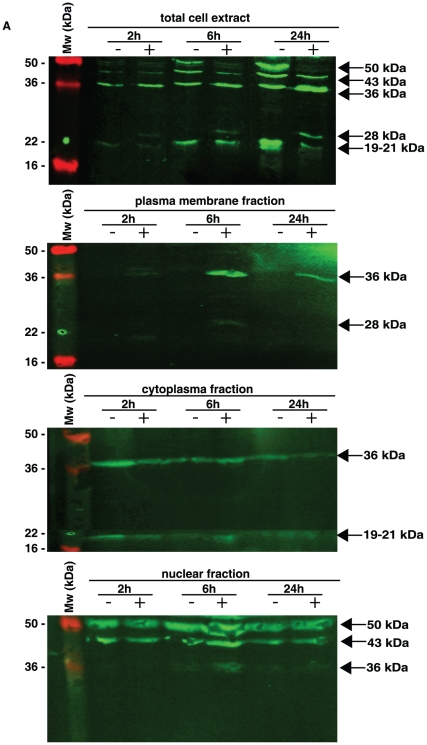
The bacterial infection induces an alternative amphiregulin cleavage pattern. Cellular fractions of control cells and infected Me-180 cells were separated by SDS-PAGE and immunoblotted. By using polyclonal antibodies against amphiregulin (MBS220633), followed by secondary IgG antibodies conjugated with IRDye 800cw, localization and distribution of amphiregulin cleavage products could be analyzed. Shown are infections for 2, 6, and 24 hours in the presence (+) or absence (−) of bacteria.

**Figure 5:**
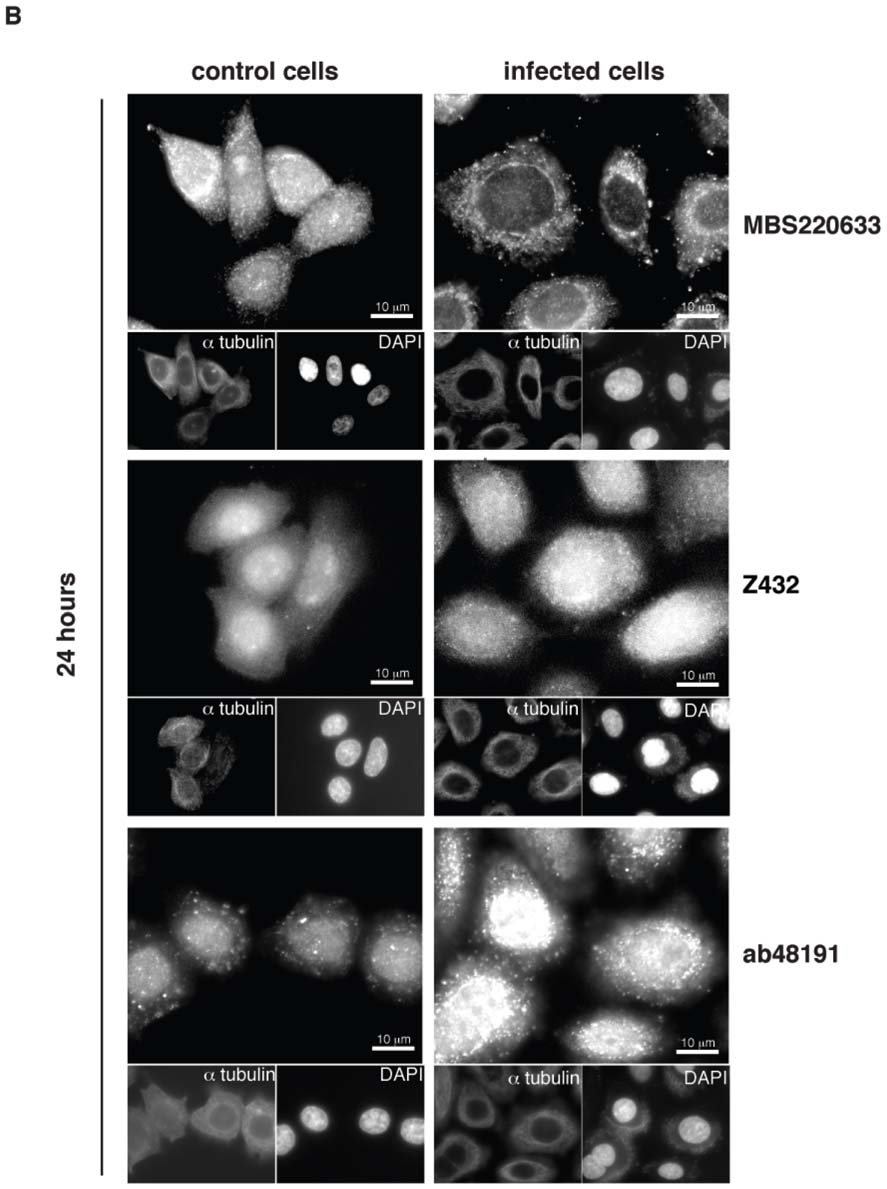
Amphiregulin protein expression in Me-180 cells. Permeabilized infected cells and control Me-180 cells stained with antibodies against different parts of amphiregulin, the bioactive domain (MBS220633), the full-length protein (Z432), and the pro-region of amphiregulin (ab48191). The binding sites for each of the antibodies used are visualized in figure 7C. Cells were control stained with α-tubulin and Hoechst 33342. Scale bars are indicated. Images are representative from 3 independent experiments.

During the different time points of *N. gonorrhoeae* infection, we clearly observed a changed pattern in subcellular localization of amphiregulin. Upon infection, amphiregulin is shuttled within the Me-180 cell in a pattern different from uninfected cells (Figure 4 and 5). Protein lysates from infected and non-infected cells were separated into fractions containing cell plasma membrane, cell cytoplasm, and cell nuclei (including nuclear membrane and nucleoplasm). The different fractions of the cells were analyzed in western blot and by using antibodies against nuclei specific Cyclin E (Figure S2) and cytoplasmic and membrane located Ezrin (Figure S3) any cross contamination of the cellular fractions could be excluded. To analyze the distribution of amphiregulin, the different subcompartments of the cells were blotted using a polyclonal antibody against the bioactive part of amphiregulin (MBS220633). In control cells, four distinct bands representing different forms of amphiregulin were detected with the sizes of 50 kDa, 43 kDa, 36 kDa, and 19–21 kDa. The 50 kDa and 43 kDa size forms of amphiregulin are also visible in the nuclear fraction, probably the nuclear membrane, while the 36 kDa and 19–21 kDa bands are found in the cytoplasm (Figure 4). Most interestingly, the 36 kDa band is present in high concentration in the plasma membrane fraction of cell lysates, only in infected cells, which correlates to previous data in figure 3. In addition, the 36 kDa form of amphiregulin is also suddenly present in the nuclear fraction (Figure 4). At the same time, there is a decrease of the full-length 50 kDa band only in infected samples, indicating that the full-length amphiregulin is more extensively processed and cleaved in infected cells.

In permeabilized and fixed Me-180 cells, amphiregulin can be detected in the plasma membrane, in the cytoplasm and in the nuclear membrane or ER when using an antibody directed against the bioactive domain (MBS220633) of amphiregulin, as visualized by immunofluorescence (Figure 5). Less amphiregulin staining was seen in the nucleus, even though having two nuclear localization sequences (NLS) within the DNA sequence [12]. After 24 hours of infection, the subcellular localization of the bioactive domain of amphiregulin was changed from the cytoplasm to the nuclear membrane. When an antibody specific against the pro-region of amphiregulin was used (ab48191), a strong and exclusive nuclear staining appears. This indicates that only the pro-region may have a reason to localize in the nucleoplasm, and an even stronger staining for nuclear localization was seen after infection. A similar pattern with stronger nuclear and cytoplasmic staining after infection was seen when using an antibody (Z432) directed against the full-length protein (Figure 5).

### Amphiregulin is not used as a receptor for initial bacterial adherence

**Figure 6:**
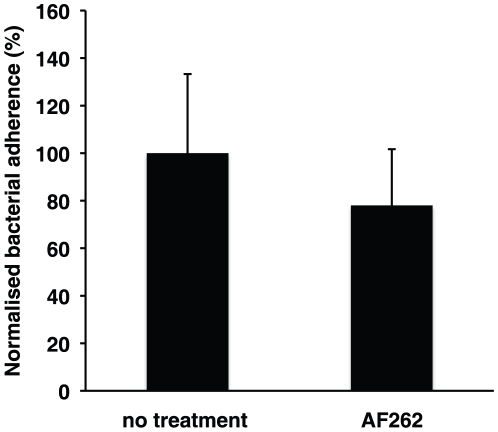
Amphiregulin is not used as a receptor for *N. gonorrhoeae*. The graph shows a bacterial binding assay to Me-180 cells. Cells were pretreated neutralizing, polyclonal antibody AF262 prior to assay. Bacterial adherence was allowed for 1.5 hours, unbound bacteria were washed away, and adherent bacteria were counted the next day. Shown is an average of 3 independent experiments and standard deviations (*P*<0.05). Percentage of bacterial adherence is presented. A 100% adherence corresponds to 40–60 adherent bacteria per Me-180 cell.

Amphiregulin is an abundant cell surface exposed glycoprotein and has the potential to play a part in bacterial binding to host cells. Also in the present paper we present data on co-localization of amphiregulin to the site of adhered bacteria. Therefore, we investigated the role of amphiregulin during initial bacterial adherence. Prior to bacterial adherence we blocked the extracellular exposed amphiregulin with polyclonal antibodies for 2 hours. These antibodies have previously been shown to neutralize the function of amphiregulin by reducing cell growth [25]. After neutralization of the amphiregulin, bacterial adherence was allowed to proceed for 90 minutes. No significant change in bacterial adherence could be detected, indicating that *N. gonorrhoeae* did not use amphiregulin as a cellular receptor for adherence (Figure 6).

Silencing amphiregulin mRNA expression by short interfering RNA (siRNA) was unsuccessful. Removal of amphiregulin resulted in growth arrest as well as reduced cell viability and no bacterial adherence (data not shown). This is in agreement with previous report where *N. gonorrhoeae* MS11 P^+^ were shown not to adhere to non-cycling cells [9].

## Discussion

Adhesion and followed invasion of *N. gonorrhoeae* to host cells is known to alter expression of a multitude of proteins in host cells. Amphiregulin has been thoroughly investigated for its role in a variety of cancers and other proliferative diseases, but not yet for its part in bacterial infections. Long-term, repeating or chronic infections of *N. gonorrhoeae* have been associated with proliferative abnormalities, although very rare [26], [27], [28]. In the present study we show that *N. gonorrhoeae* infection changes the fate of one of the most abundant of growth factors; amphiregulin. Early bacterial infection induces amphiregulin mRNA upregulation, alternate processing and cleavage, subcellular localization and release. The effects seen are prolonged for at least 24 hours of infection.

**Figure 7:**
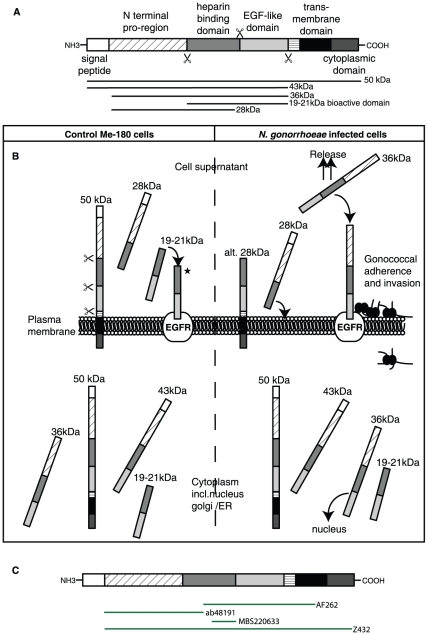
*N. gonorrhoeae* induces alternative processing and release of amphiregulin. (**A**) Pro-amphiregulin is synthesized by 252 amino acids (50 kDa). The protein consists of a signal peptide, a N-terminal pro-region, a heparin binding domain, an EGF-like domain, a transmembrane domain, and a cytoplasmic domain. Due to metalloprotease dependent cleavage, amphiregulin can produce several products with the molecular sizes 50, 43, 36, 28, and 19–21 kDa. (**B**) Schematic illustration of amphiregulin expression, cleavage pattern and processing in Me-180 cells. *N. gonorrhoeae* infection changes the cleavage pattern, subcellular localization, processing, and release of amphiregulin in Me-180 cells. (* Indicates alternative fate of cleaved amphiregulin [11].) (**C**) Illustration of the recognition sites of the antibodies used in this study.

Amphiregulin is synthesized as a 50 kDa (252 amino acids) pro-peptide (Figure 7A). At the plasma membrane, it undergoes proteolytic cleavage by a specific member of the ADAM family of proteases, ADAM17, to produce the bioactive domain [10]. The bioactive domain is a 19–21 kDa (78 or 84 amino acids) product containing a heparin binding domain and an epidermal growth factor (EGF) like domain (Figure 7A). After binding of the bioactive domain to its autocrine receptor, EGFR, additional events occurs, such as auto-phosphorylation, internalization, endocytosis and recycling of the receptor [29]. Several predicted models of the cleavage and release of amphiregulin have been proposed, and it seems to depend on cell line analyzed and external conditions [11], [14], [30].

Here, we propose an adapted model for the amphiregulin protein processing in Me-180 cells and how it changes during *N. gonorrhoeae* infection (Figure 7B). The parts of the protein to which the different antibodies are directed against, are also illustrated in Figure 7C. In uninfected control Me-180 cells, amphiregulin is localized to the cytoplasm, nuclear membrane or ER and a small amount is also found on the plasma membrane. The main localization of the bioactive protein was observed in the cytoplasm in accordance with previous studies in other cell lines [16], [31], [32]. The cytoplasmic location of bioactive amphiregulin could be explained by ligation of the receptor that upon binding is internalized and recycled [29]. In western blot of control cells amphiregulin was seen as four distinct bands of 50 kDa, 43 kDa, 36 kDa and 19–21 kDa in size. Upon infection an additional band of 28 kDa appeared. The different domains and cleaved fragments of amphiregulin have separate fates after shedding from the membrane and exert different functions on cellular processes. The larger band of 43 kDa corresponds to the heparin binding domain and EGF domain in connection with the N-terminus. It is seen in the nuclear fraction while the lower bands at 36 kDa found in the cytoplasm represent the same fragment after removal of the signal peptide. There is also a constant release of a 28 kDa product into the supernatant. We propose that this 28 kDa band is represented by the heparin binding domain in connection with the pro N-terminus (Figure 7B). In the uninfected cells we do not detect any protein in the plasma membrane fraction on the western blot even though we see a signal in the fluorescence microscope. This could be to the fact that only a little protein is found on the surface in un-stimulated Me-180 cells. The bioactive cleavage product of 19–21 kDa that could be responsible for EGFR activation on the plasma membrane, even though other soluble factors of amphiregulin containing the EGF domain could also be involved in this process [11]. In Me-180 cells, we observed a strong signal in fluorescence microscopy for the N-terminus of amphiregulin in the nucleoplasm, which was not seen for any of the other domains. The N-terminus of pro-amphiregulin has been documented to be required for proper folding and export from the cell since deleted N-terminal pro-region completely abolished the release of amphiregulin [30].

After understanding amphiregulin in uninfected control Me-180 cells, we then investigated amphiregulin during bacterial infection in three different cell lines. We show that the infection by *N. gonorrhoeae* MS11 P^+^ immediately enhances the transcription of amphiregulin in ME-180 cells and HEC-1-B cells, but not in VK2/E6E7 cells. The minor increase in *N. lactamica* and *E. coli* infected cells may be due to toll-like receptor 4 activation via bacterial lipooligosaccharide (LOS) expression, as has been reported for intestinal cells [33]. Even though, this is not in agreement with previous findings where LOS expressing *N. meningitidis* did not increase the transcription of amphiregulin after 6 hours of infection [23]. Already after one hour of infection with MS11 P^+^ in Me-180 cells, the mRNA levels increase to high levels. But a following increase in total protein concentrations does not occur inside the cell. Upon infection, the majority of the full-length 50 kDa pro-amphiregulin is rather cleaved immediately into a 36 kDa product, exclusively localized to the cytoplasm and nuclear fraction as determined by western blot. As a consequence, reduced protein levels of the pro-amphiregulin can be seen. In addition, these 36 kDa products are released to the cell culture supernatant and bind to the plasma membrane, most likely to the EGFR in the infected cells. Amphiregulin functions in an autocrine manner and the bioactive form (19–21 kDa cleavage product) is known to bind the EGFR on the outside of the plasma membrane. An increased release would therefore lead to more amphiregulin bound to the surface of the Me-180 cells that in turn leads to a stimulatory response in the cell to upregulate amphiregulin at 6 hours post infection. As expected, an increased fluorescent staining of plasma membrane bound amphiregulin in non-permeabilized cells can be seen after 6 hours of infection. The staining co-localized with the site of bacterial adherence, which further proves the binding of amphiregulin to the EGFR, previously shown to cluster at the cortical plaque formations underneath the site of adhered bacteria [3]. There is also an appearance of the 28 kDa factor on the membrane after bacterial adherence, possibly being the released product binding to the plasma membrane with its heparin binding domain via heparan sulfate glycosaminoglycan, which has been shown to act as a co-receptor for amphiregulin [34]. Alternatively, the 28 kDa band could be a membrane bound bioactive protein containing also the transmembrane and cytoplasmic tail domains, formed upon infection (Figure 7B). Binding of gonococci to cell surface heparan sulfate proteoglycans has previously been shown to have a role in the establishment of infection and a member of the opacity (opa) protein family in *N. gonorrhoeae* mediates this attachment. This interaction could be of interest to study further, as it could be part also of an explanation for the co-localization at the membrane, but has not been the focus of this work where an opa-negative strain has been used.

The bi-functional role of amphiregulin as a growth regulator in Me-180 cells during *N. gonorrhoeae* infection will be under further investigation. Interfering with host epithelial layer probity and modifying cellular growth at the cervical mucosa will most likely favor bacterial infection, intracellular survival and disease progression. A cell with impaired growth reduces both the protection of the mucosal barrier, its integrity and repair mechanisms as well as inflammatory responses and defense mechanisms. The data presented in this work highlights the importance of studying the role of the pathogenic bacterium *N. gonorrhoeae* in the regulation of host cell growth and proliferation by modifying targets such as the major epidermal growth factor amphiregulin.

## Materials and Methods

### Cell line and bacterial strain

Me-180 (ATCC HTB33) epithelial-like cell line from cervical carcinoma was maintained in DMEM supplemented with 10% inactivated fetal bovine serum (FBS) (Invitrogen) at 37°C, 5% CO_2_. HEC-1-B (ATCC HTB 113) human endometrial adenocarcinoma cell line were maintained in Eagle minimal essential medium supplemented with 10% FBS at 37°C, 5% CO_2_. VK2/E6E7 (ATCC CRL-2616) human epithelial vaginal cells were maintained in keratinocyte-serum free medium (Invitrogen) supplemented with 0.1 ng/ml human recombinant EGF, 0.05 mg/ml bovine pituitary extract and 44.1 mg/l calcium chloride, all purchased from Invitrogen. *N. gonorrhoeae* adheres and invades VK2/E6E7 cells [35]. All cell lines were sub-cultured 2–3 times per week and never grown to high confluence. Cells were always sub-cultured 18–24 hours prior assays. *Neisseria gonorrhoeae* strain MS11mk (P+) [36] is referred in the text to MS11 P^+^. Piliated, Opa-negative phenotypes were used in assays, as distinguished by colony morphology and opacity under binocular light microscope. The *N. gonorrhoeae* MS11 P^−^n variant harbors a deletion at the 5′ end of *pilE* gene resulting in a non-piliated phenotype [36]. Non-pathogenic *N. lactamica* properties have been described previously [37]. The bacteria were grown on GC medium base (Difco) agar plates containing Kellogg's supplement [38] at 37°C, 5% CO_2_. Piliated, Opa-negative bacteria were selected and sub-cultured every 18–24 hours. The *E. coli* strain DH5α were grown on LB agar plates (BD Biosciences) at 37°C and 5% CO_2_.

### Bacterial infection of cells

Upon assay, 18–24 hours old bacteria were harvested from the plates in sterile phosphate buffered saline (PBS). The number of bacteria was calculated by measuring the absorbance at 600 nm wavelengths. Optical density was adjusted to 1, which equals 5×10^8^ bacteria per milliliter. Bacteria were added to a non-confluent monolayer of Me-180 cells at a multiplicity of infection (MOI) of 100. The infection was allowed to proceed for 1–72 hours in 37°C, 5% CO_2_. For longer assays, unbound bacteria were carefully washed away with DMEM after 2 hours, 10 hours and 24 hours to prevent overgrowth in the cell culture medium. Through out all experiments, uninfected control cells were treated equally in terms of incubation and washing procedures, ensuring that the only difference between the control and samples were specific to the gonococcal infection.

### Quantitative real time PCR analysis

RNA from control cells and infected Me-180 cells was isolated with the RNeasy Mini kit according to manufacturers recommendations (Qiagen). Total RNA (up to 1 µg) was reverse transcribed using Superscript III First strand synthesis supermix (Invitrogen), using oligo-dT primers according to manufacturers recommendations. Primers used were: Amphiregulin (forward 5′-GTGGTGCTGTCGCTCTTGATACTC-3′ and reversed 5′-TCAAATCCATCAGCACTGTGGTC-3′), EGFR (forward 5′- ACTGCACCTACGGATGCACTGG-3′ and reversed 5′- AACGATGTGGCGCCTTCGCA-3′), and GAPDH (forward 5′TCGTCATGGGTGTGAACCATGAGA-3′, reversed 5′TGTGGTCATGAGTCCTTCCACGAT-3′). PCR amplification was performed using a LightCycler 480 (Roche) and SYBRGreenI Master kit (Roche) with 0.1 µM of each gene specific primer and 1 µl of cDNA. PCR program was as follows: initial denaturation for 300 s, amplification for 40 cycles with denaturation at 95°C for 20 s, annealing at 60°C for 15 s and extension at 72°C for 15 s. The transition rate was 2.2–4.4°C/s. Melting curve analysis was conducted in three segments: 95°C for 5 s, 70°C for 60 s and then increasing to 95°C. The transition rate was 2.2–4.4°C/s for the first and second segment and 0.19°C/s for the last segment. Concentrations of mRNA of amphiregulin and EGFR were analyzed and compared, using GAPDH as an internal standard. Expression ratios were calculated, normalized, and compared between control cells and infected cells.

### Antibodies

The monoclonal antibody Z432 (Santa Cruz) was raised against amphiregulin full-length protein and was used in immunofluorescent assays with a concentration of 10 µg/ml (exact binding position is not known). The polyclonal antibody ab48191 (Abcam) against the N-terminal region of the pro-peptide amphiregulin was used in immunofluorescent assays with a concentration of 3 µg/ml. The polyclonal antibody MBS220633 (Mybiosource) was raised against amino acids 108–126 of the bioactive domain of amphiregulin. MBS220633 was used at concentrations of 20 µg/ml for western blotting, immunofluorescence, ELISA assay and flow cytometry. The polyclonal neutralizing and inhibiting antibody AF262 (R&D systems) was raised in goat against amino acid 101–198 of amphiregulin. AF262 was used at the concentration of 25 µg/ml and has previously show good neutralizing capacities [25]. The Secondary antibodies goat-anti mouse IgG –Alexa 488 or goat-anti rabbit IgG –Alexa 488 was used at concentrations; 2–4 µg/ml for immunofluorescence and flow cytometry. In western blot, secondary antibodies goat-anti mouse IgG –IRDye 800 cw (Licore) or goat-anti rabbit IgG –IRDye 800 cw (Licore) were used at concentrations 0.1 µg/ml. In ELISA, secondary antibodies goat-anti rabbit IgG –HRP (Bio-Rad) was used at concentrations 0.4 µg/ml. None of the antibodies directed against amphiregulin cross-reacted against any bacterial proteins. Control immunoblots were made of the cell fractionations using a monoclonal antibody against Ezrin (sc-32759, Santa Cruz) at a concentration of 0.4 µg/ml and a monoclonal antibody against Cyclin E (HE-12, Zymed) at 0.5 µg/ml. Control immunofluorescent stainings were made by rabbit anti α-tubulin antibodies (MBS316320, Mybioscource) at 30 µg/ml or mouse anti α-tubulin antibodies (TUB-1A2, Sigma) 50 µg/ml.

### Fluorescence microscopy

Me-180 cells were cultured on 1% metasilicate coated cover glass slips (Merck, 13 mm, 0.17 mm thick) over night to produce 50–60% of confluence. Cells were infected with a MOI of 100 of *N. gonorrhoeae* MS11 P^+^ for 6 hours and 24 hours. Cells were fixated with 4% paraformaldehyde (Sigma) for 10 minutes in room temperature, washed with PBS, and permeabilized for 5 minutes in 0.1% NP-40, 20 mM TrisHCl (pH 7.4), 50 mM NaCl, 300 mM Sucrose, and 3 mM MgCl_2_. Then, cells were washed carefully in PBS and blocked with 3% bovine serum albumin (BSA). Cells were stained with the different antibodies against amphiregulin for 45 minutes. Secondary antibodies were added for 45 minutes after washing three times with PBS containing 0.01% Tween 20 (Sigma). Non-permeabilized cells were fixated with 4% paraformaldehyde (Sigma) for 10 minutes in room temperature, washed with PBS (no Tween 20 added). Glasses were mounted in Vectashield containing Hoechst 33442 DNA stain (Vector laboratories Inc, Burlingame, CA, USA). The cells were imaged using a Zeiss fluorescence microscope.

### ELISA

Cells were infected with a MOI of 100 of *N. gonorrhoeae* MS11 P^+^ for 1, 2, and 6 hours, or with *N. gonorrhoeae* MS11 P^−^n, *N. lactamica*, or *E. coli* for 1 and 6 hours (MOI 100). Cell culture supernatants from uninfected and infected cells were collected and treated with Complete mini protease inhibitors mix (Roche) to avoid protein degradation. DMEM treated the same way and with or with out added bacterial suspension, were used as controls to exclude any possible cross reactions with bacterial products. Immunoplates (Maxisorp 96 well, Nunc) were coated in triplicates with 0.1 ml samples (diluted in carbonate buffer) at 4°C over night. The wells were blocked with 0.2 ml 1% BSA (Sigma) for 2 hours, washed and amphiregulin was detected with the antibody MBS220633 against amphiregulin (diluted in 1% BSA in PBS) followed by incubation with HRP conjugated anti-IgG antibodies. For enzymatic color development, 0.1 ml TMB (Invitrogen) was added and then 1 M HCl to stop the reaction. The absorption was read at 450 nm wavelengths.

### Western blot analysis

Cells were infected with a MOI of 100 of *N. gonorrhoeae* MS11 P^+^ for 2, 6, and 24 hours. Infected cells and control cells were carefully washed three times in PBS and then harvested on ice by addition of SDS reducing sample buffer (87% glycerol, 10% SDS, 0.5 M Tris-HCl pH 6.8, 0.1% bromphenol blue) and immediately stored at −20°C until use. Samples were incubated at 95°C for 10 min and 5 µl was loaded onto a 12% gel for SDS-PAGE according to manufacturers recommendations (Bio-Rad) After separation, proteins in the gel were transferred to PVDF membranes (Milli-Pore) using semidry transfer system (Bio-Rad). The membranes were washed in water and blocked overnight at 4°C in 5% nonfat dry milk (Bio-Rad) diluted in PBS. The membranes were incubated in room temperature, agitating with the MBS220633 antibody against amphiregulin for 45 min. Incubation with secondary antibody goat anti-rabbit IRDye 800cw was performed for 45 min. Bands were visualized using Odyssey infrared imaging system (Li-Core).

### Flow cytometry assay

Cells were infected with a MOI of 100 of *N. gonorrhoeae* MS11 P^+^ for 6 hours. Infected cells and control cells were carefully washed three times in PBS and then harvested by trypsination (Trypsin/EDTA, Invitrogen). Cells were washed in PBS by centrifugation at 1000× g for 10 minutes, and fixed with 4% paraformaldehyd for 10 minutes in room temperature. Cells were stained with the antibody MBS220633 against amphiregulin for 45 minutes. Secondary Alexa 488 nm conjugated antibodies were added for 45 minutes after washing three times with PBS. The flow cytometry device counted 10 000 cells for fluorescence (FACScan).

### Cell fractionations

Cells were infected with a MOI of 100 of *N. gonorrhoeae* MS11 P^+^ for 2, 6, and 24 hours. Infected cells and control cells were carefully washed three times in PBS and then scraped off the culture dish and collected in an eppendorf tube. The cells were centrifuged at 1000× g for 10 minutes at 4°C and the pellets were permeabilized in 100 µl 0.2% Igepal/protease inhibitor mix/PBS on ice for 5 minutes. The suspension was forced through a pipette tip to break the membranes. The nuclear fraction was separated from the membrane and cytoplasm by centrifugation at 1000× g for 10 minutes at 4°C. Spinning at 110× g for 30 minutes at 4°C separated the membrane and cytoplasmic fractions. The nuclear fraction was once washed in cold PBS and centrifuged at 2900× g for 10 minutes at 4°C. The nuclei were resuspended in cold PBS with a 29-gauge needle to break the nuclear membranes. The solution was treated with DNase (Promega) for 5 minutes at room temperature. SDS reducing sample buffer was added to all fractions and samples were stored at −20°C immediately until further use.

### Bacterial adherence assay

Cells were infected with a MOI of 100 of *N. gonorrhoeae* MS11 P^+^ for 2 hours. Infected cells and control cells were carefully washed three times in PBS and bacteria were harvested by addition of 1% saponin for 5 minutes, serially diluted and spread onto GCB-plates. Plates were incubated over night at 37°C, 5% CO_2_ and colony forming units (c.f.u.) were counted the following day. Antibody inhibition of amphiregulin was performed by a pre incubation of cells in 25 µg/ml of an inhibiting amphiregulin antibody raised in goat (AF262) for 1.5 hours prior addition of bacteria.

### Statistical analysis

Released AREG in ELISA assay and bacterial adherence assays were evaluated using a two-tailed Student's *t* test. Values of *P*<0.05 were considered significant. For quantitative PCR analysis, One-way ANOVA (*P*<0.05) (VasserStats, NY, U.S.A) was used to compare values between infected cells and uninfected control cells.

## Supporting Information

Figure S1 **Immunoblot of cell supernatants after 6 hours of infection.** Western blots of ME-180 cell supernatants (upper membrane), HEC-1-B cell supernatants (middle membrane), and VK2/E6E7 cell supernatants (lower membrane) from 6 hour of incubation in the presence of different bacterial strains.
(EPS)

Figure S2 **Cyclin E is localised in the nucleus fraction.** Control immunoblot of cell fractionations. Fractions of Me-180 cell membrane, cytoplasm and nuclei were separated by SDS-PAGE and immunoblotted using antibodies specific against proteins expressed in these cell compartments. Secondary antibodies goat-anti IgG IRDye 800cw were used. Cyclin E is expressed in the nucleus.
(EPS)

Figure S3 **Ezrin is localised in the cytoplamic fraction.** Control immunoblot of cell fractionations. Fractions of Me-180 cell membrane, cytoplasm and nuclei were separated by SDS-PAGE and immunoblotted using antibodies specific against proteins expressed in these cell compartments. Secondary antibodies goat-anti IgG IRDye 800cw were used. Ezrin is expressed in the cytoplam.
(EPS)
